# Daple deficiency causes hearing loss in adult mice by inducing defects in cochlear stereocilia and apical microtubules

**DOI:** 10.1038/s41598-021-96232-8

**Published:** 2021-10-12

**Authors:** Yoshiyuki Ozono, Atsushi Tamura, Shogo Nakayama, Elisa Herawati, Yukiko Hanada, Kazuya Ohata, Maki Takagishi, Masahide Takahashi, Takao Imai, Yumi Ohta, Kazuo Oshima, Takashi Sato, Hidenori Inohara, Sachiko Tsukita

**Affiliations:** 1 Laboratory of Biological Science, Graduate School of Frontier Biosciences and Graduate School of Medicine, Osaka University, 2-2 Yamadaoka, Suita, Osaka 565-0871 Japan; 2 Department of Otorhinolaryngology-Head and Neck Surgery, Graduate School of Medicine, Osaka University, 2-2 Yamadaoka, Suita, Osaka 565-0871 Japan; 3 Department of Pharmacology, Teikyo University, 2-11-1 Kaga, Itabashi, Tokyo 173-8605 Japan; 4 Advanced Comprehensive Research Organization (ACRO), Teikyo University, 2-21-1 Kaga, Itabashi, Tokyo 173-0003 Japan; 5 Department of Biology, Faculty of Mathematics and Natural Science, Universitas Sebelas Maret, Jalan Ir. Sutami 36 A, Surakarta, 57126 Indonesia; 6 Department of Neuroscience and Cell Biology, Graduate School of Medicine, Osaka University, 2-2 Yamadaoka, Suita, Osaka 565-0871 Japan; 7 Department of Pathology, Graduate School of Medicine, Nagoya University, 65 Tsurumai, Showa, Nagoya, Aichi 466-8550 Japan; 8 International Center for Cell and Gene Therapy, Fujita Health University, 1-98 Dengakugakubo, Kutsukake, Toyoake, Aichi 470-1192 Japan

**Keywords:** Cell biology, Cell polarity, Developmental biology, Morphogenesis

## Abstract

The V-shaped arrangement of hair bundles on cochlear hair cells is critical for auditory sensing. However, regulation of hair bundle arrangements has not been fully understood. Recently, defects in hair bundle arrangement were reported in postnatal Dishevelled-associating protein (ccdc88c, alias Daple)-deficient mice. In the present study, we found that adult *Daple*^−/−^ mice exhibited hearing disturbances over a broad frequency range through auditory brainstem response testing. Consistently, distorted patterns of hair bundles were detected in almost all regions, more typically in the basal region of the cochlear duct. In adult *Daple*^−/−^ mice, apical microtubules were irregularly aggregated, and the number of microtubules attached to plasma membranes was decreased. Similar phenotypes were manifested upon nocodazole treatment in a wild type cochlea culture without affecting the microtubule structure of the kinocilium. These results indicate critical role of Daple in hair bundle arrangement through the orchestration of apical microtubule distribution, and thereby in hearing, especially at high frequencies.

## Introduction

Apical differentiation of epithelial cell sheets is critical for the functioning of organs. In the inner ear, the differentiation of stereociliary hair bundles in cochlear hair cells (HCs) is essential for hearing. All hair bundles in the cochlea form V-shaped vertices oriented in the same abneural (lateral) direction. Planar cell polarity (PCP) coordinates the alignment of cell polarities across a tissue plane at a cell-to-tissue level^1,2^. However, our knowledge regarding the mechanisms underlying this coordination remains fragmentary. Dishevelled (Dvl)-associated protein (ccdc88c, alias Daple), with a high leucine content, was first identified as a scaffold protein that interacts with Dvl, a core PCP protein^3^. Daple has been reported to regulate several biological activities, such as cell differentiation, proliferation, morphology, and cancer cell dynamics, at least partially through Frizzled-Gαi-related Wnt signaling^4–7^. Recently, deletion of the Ccdc88c gene (hereafter referred to as Daple) was shown to cause defects in the arrangement of hair bundles in the HCs of the organ of Corti (OC) in mice^8^. In *Daple*^−/−^ mice, the dissociated localization of Gαi proteins and the primary cilium of HCs, called kinocilium, has been reported to occur during the neonatal period and causes defects in the arrangement of hair bundles^8^. However, the hearing potential of mature cochlea was not analyzed in these mice. Furthermore, the multifaceted molecular mechanisms that connect deletion of *Daple* and defects in the arrangement of stereocilia remain at least partly elusive. Like *Daple*^−/−^ mice, mice deficient in Lis-1, a dynein regulatory protein also exhibit apical morphological deformities in HCs in the OC^9^, suggesting the involvement of microtubules in the formation of apical structures in the HCs of the OC in the context of PCP. However, the role of apical microtubules in apical morphogenesis in HCs of the OC remains to be elucidated.

In this study, we analyzed Daple-deficient mice, from neonatal to adult stages, to determine the role of Daple in HC apical morphogenesis, especially via microtubules. We show the presence of hearing disturbances at all frequencies examined using the auditory brainstem response (ABR) test, especially at high frequencies. Reflecting the ABR results, malformation of hair bundles was found to be more severe in the basal area, indicating that Daple plays a consistent role in the cochlea for hearing. Our findings also unravel the role of apical microtubules in HC apical differentiation, which is consistent with the results obtained upon nocodazole administration. Finally, Daple seems to be essential, especially during the morphogenesis of hair bundles, because malformation of hair bundles was consistent from birth to adulthood in Daple-deficient mice.

## Results

### Auditory brainstem response (ABR) testing revealed hearing defects in Daple^−/−^ adult mice, especially at higher frequencies

**Figure 1:**
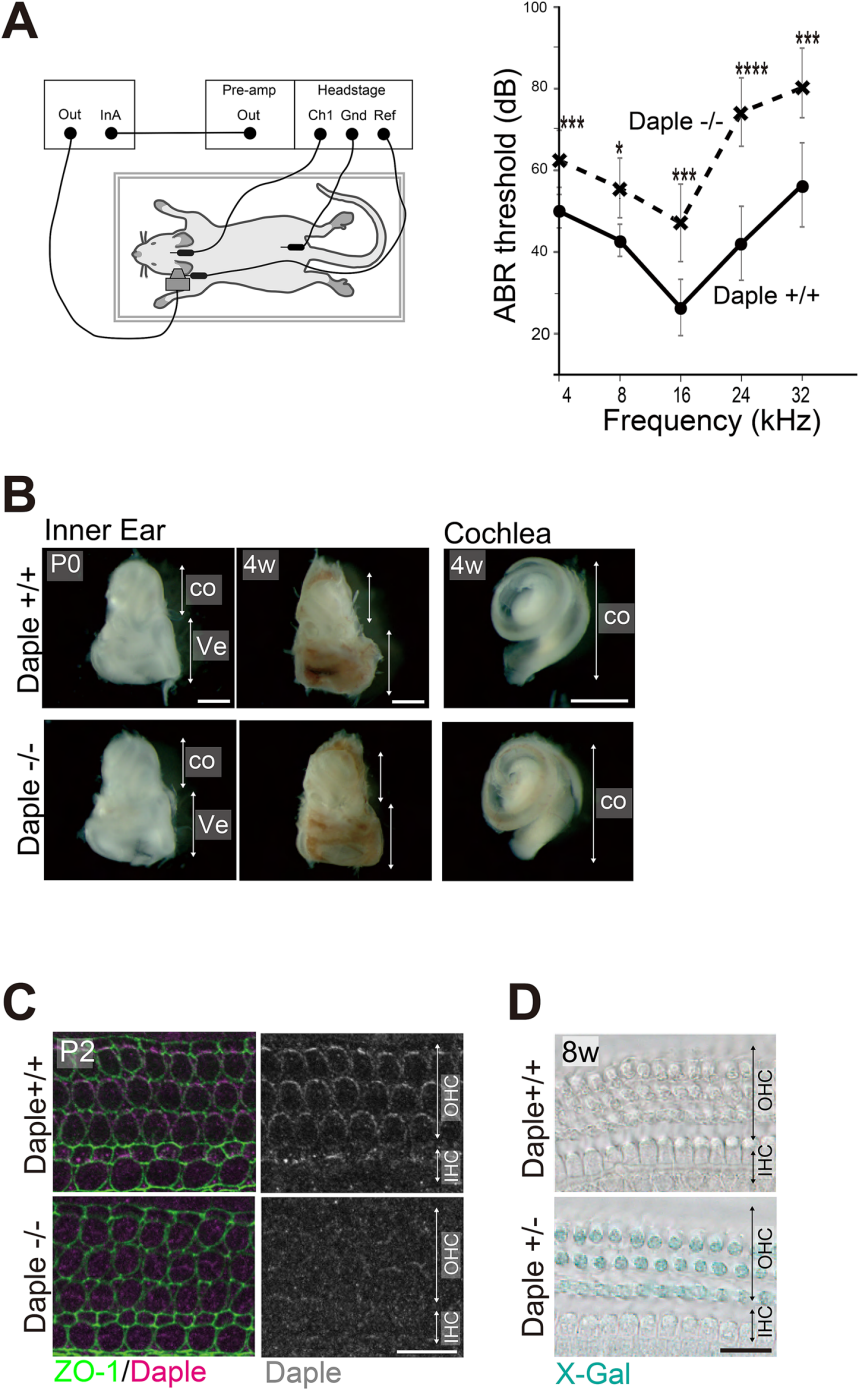
Hearing ability, inner ear morphology, and Daple expression in Daple-deficient mice. (**A**) The auditory brainstem response (ABR) thresholds of *Daple*^−/−^ mice (8–12-week-old, n = 7) were significantly higher than those of *Daple*^+/+^ mice (8–12-week-old, n = 7; *p* < 0.05). At around 24 kHz, ABR thresholds were significantly higher than in control mice (*p* < 0.003). *p < 0.05, ***p < 0.005, ****p < 0.003. (**B**) The gross shape and length of the cochlear duct in the inner ears of *Daple*^+/+^ and *Daple*^−/−^ mice (postnatal day (P)0, 4-week-old) seemed normal. (**C**) Immunostaining of the OC in wild-type P2 HCs from middle cochleae. Daple was stained on the lateral side of OHCs and IHCs. Additionally, some staining of centrioles was evident in HCs. The enrichment was not visible in *Daple*^−/−^ littermates. HC, hair cell; OHC, outer hair cell; IHC, inner hair cell. (**D**) Daple promotor activity could be detected by the intensity of X-Gal staining as the LacZ gene was expressed as a fusion protein with exons 1–3 of Daple gene in Daple + /- mice. LacZ-Xgal staining revealed expression patterns of *Daple* in adult *Daple*^+/−^ and *Daple*^+/+^ mice from middle cochleae. *Daple*^+/−^ OHCs are stained blue. Scale bars: B; 1 mm, C; 10 μm, D; 20 μm.

Although *Daple*^−/−^ mice, in the embryonic and neonatal stages, have previously been reported to have defects in the arrangement of hair bundles in cochlear HCs^8^, hearing-potential and morphological changes with age have not been analyzed. Here, we performed ABR tests on 8–12-week-old mice and found lower sensitivity to sounds in *Daple*^−/−^ mice than in *Daple*^+*/*+^ mice at all frequencies, ranging from 4 to 32 kHz. A highly significant hearing disturbance was detected around 24 kHz (42.1 ± 9.1 in ^+/+^ vs. 74.3 ± 8.4 dB in ^−/−^, *p* < 0.003, Fig. 1A). The gross shape and size of the cochleae in *Daple*^−/−^ mice were comparable to those of *Daple*^+/+^ mice (Fig. 1B), which is consistent with a previous report regarding neonatal cochleae^8^. In addition, we found that the gross shape and size of the cochleae in *Daple*^−/−^ mice were also similar to those of the cochleae in *Daple*^+/+^ mice at 4 weeks of age. These results suggested that sound wave transmission along the snail-shaped OC from the tympanic membrane occurs in the same way in the cochleae of both *Daple*^+/+^ and *Daple*^−/−^ mice^10^. As localization and expression of Daple was similar between neonates and adults, along the apex to the base, in the cochlea (Fig. 1C,D; Supplementary Fig. S1), the hearing disturbances observed at all sound frequencies in Daple^−/−^ mice are reasonable, although disruption at higher frequencies suggested the presence of certain functional and/or structural failures involving HCs in the more basal regions of the OC.

### Hair bundle arrangement was affected in adult Daple^−/−^ mice, especially in the basal region

**Figure 2:**
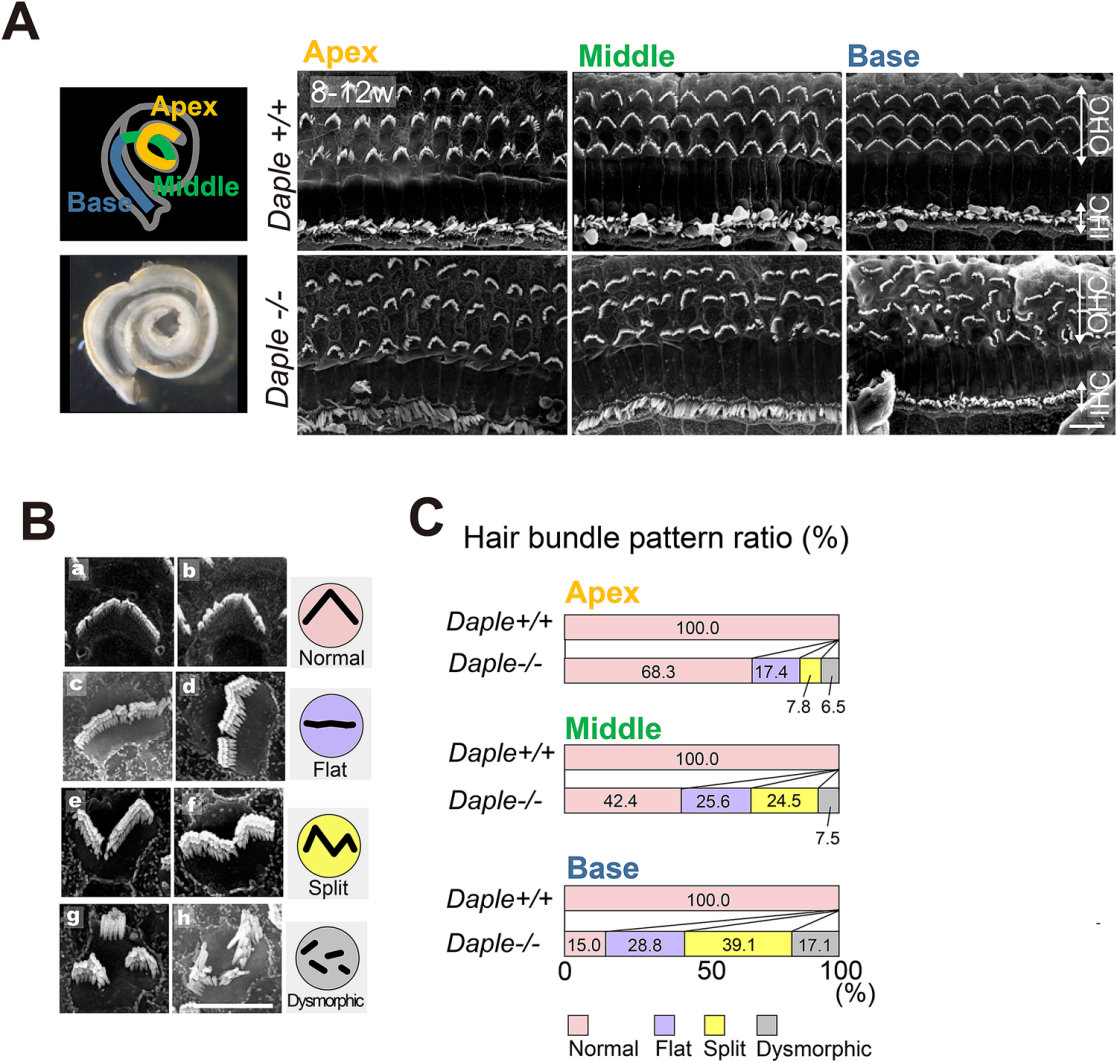
Scanning electron microscopy (SEM) analysis of Daple-deficient organ of Corti (OC) in adult mice. (**A**) SEM images of hair cells (HCs) from apex, middle, and basal areas of *Daple*^+/+^ and *Daple*^−/−^ cochleae in adult mice. *Daple*^−/−^ cochleae exhibit major abnormalities in hair bundles in OHCs, high magnification images of OHCs. (**B** a, b) Daple-/- HCs with normal bundles. (**B** c, d) *Daple*^−/−^ HCs with flat bundles (jagged horizontal line). (**B** e, f) *Daple*^−/−^ HCs with split bundles (reversed apex region of the V-shape bundle). (**B** g, h) *Daple*^−/−^ HCs with a generally deformed bundles (dysmorphic bundle; fragmented bundle). Scale bars: A; 10 μm, B–G; 5 μm. (**C**) Analysis of the difference between Daple^+ / +^ and *Daple*^−/−^ mice in the three cochlear areas (apex, middle, and basal areas), (8–12-week-old, Daple + / + apex 91 cells, middle 95 cells, and basal 94 cells, *Daple*^−/−^ apex 632 cells, middle 477 cells, and basal 455 cells). The ratios of Daple-deficient mouse HCs tended to increase with some deformed bundles.

Based on the abovementioned observations, we next examined the possible associations between the ABR results and morphological defects in hair bundles in adult *Daple*^−/−^ mice, aged 8–12 weeks using scanning electron microscopy (SEM). In adult mouse outer hair cells (OHCs), deformities were observed in the hair bundles (Fig. 2A), along with various morphological alterations similar to those observed in prior studies on neonatal *Daple*^−/−^ mice^8^; these were classified as follows: normal (Fig. 2B a,b), flat (Fig. 2B c,d), split (Fig. 2B e,f), and other dysmorphic bundles (Fig. 2B g,h). Although defects in the arrangement of hair bundles were observed over the entire length of the cochlea, the number of cells with defects in hair bundles was higher in the more basal cochlear regions in *Daple*^−/−^ mice (% ratios of each dysmorphic bundle type from the apex to the base region, respectively: 17.4, 25.6, 28.8 in flat; 7.8, 24.5, 39.1 in split; 6.5, 7.5, and 17.1, respectively) (Fig. 2C). In contrast, in *Daple*^+/+^ mice, almost all the HCs exhibited normal V-shaped bundles (Fig. 2C *Daple*^+/+^; apex 91 cells, middle 95 cells, base 94 cells). The increase in the ratio of abnormal hair bundle arrangement cells from 32% in the apex to 58% in the middle to 85% in the base suggested an association between the ABR results and morphological defects in hair bundles in 8–12-week-old adult *Daple-*deficient mice.

### Deformed apical structures, comparable to those in 8–12-week-old adult mice, were present in postnatal day (P)3 Daple^−/−^ mice

**Figure 3:**
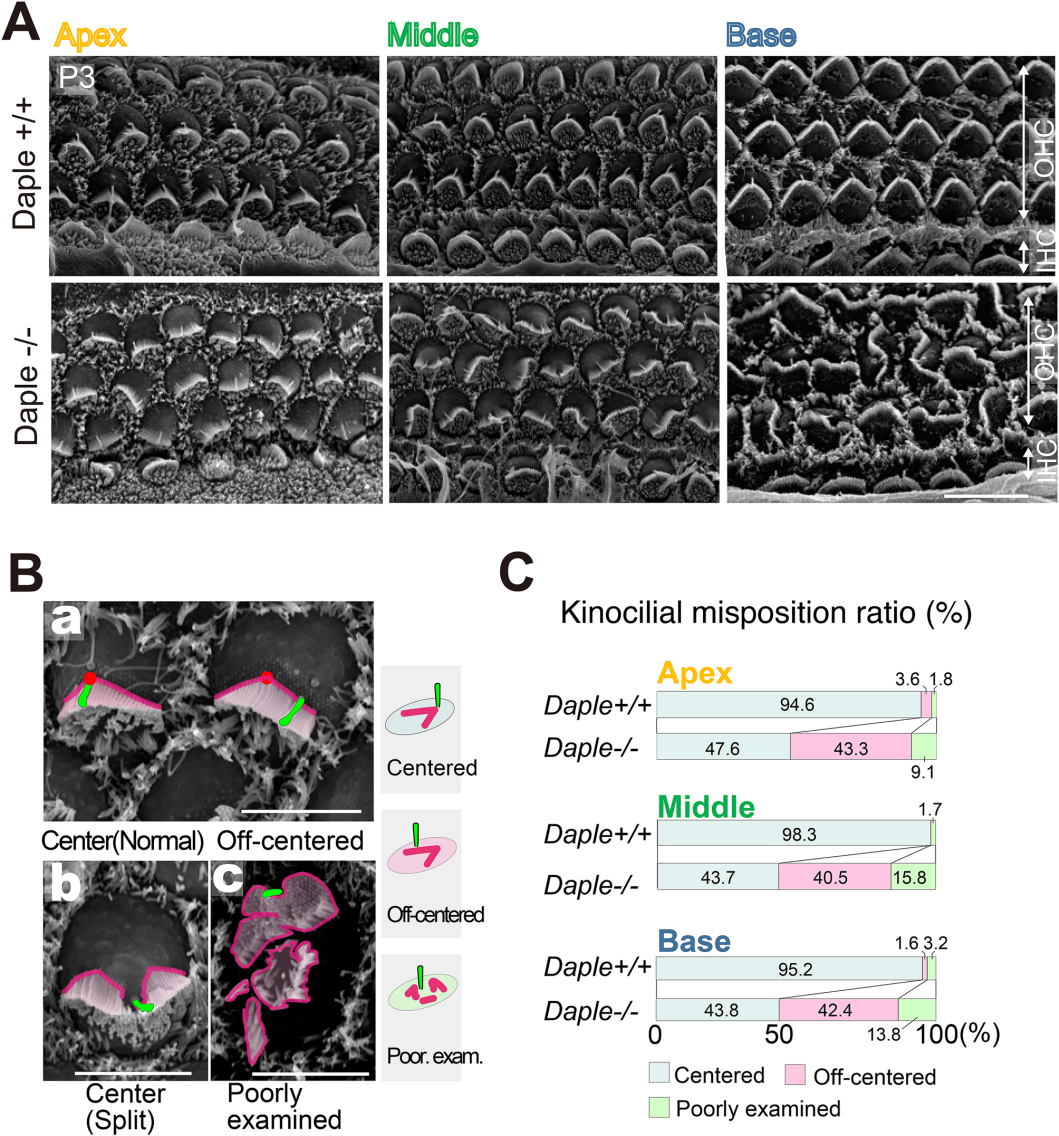
Analysis of Daple-deficient organ of Corti (OC) in mouse pups. (**A**) Scanning electron microscopy images of hair cells (HCs) from the apex, middle, and basal areas of *Daple*^+/+^ and *Daple*^−/−^ cochleae in P3 mice. The most severe effects of Daple deficiency were observed in the basal areas of cochlea. (**B**a) Normal V-shaped bundles in *Daple*^−/−^ mice: left HC showed normal kinocilium locations and right HC showed abnormal kinocilium locations. (**B**b) Split and (**B**c) poorly examined bundles: the stepwise arrangement of stereocilia bundles was missing. (**C**) We classified the localization of kinocilia against hair bundles into three groups: normal (centered), off-centered, and poorly examined. When we could not define the kinocilia, we classified them into the poorly examined group. Over half of *Daple*^−/−^ kinocilia in all three areas are off-centered (at P3, n = 5; apex 691 cells, middle 886 cells, basal 765 cells). Scale bars: (**A**); 10 μm, (**B**)–(**D**); 5 μm.

To compare morphological changes in hair bundles with age, we next focused on the cochleae of neonatal mice. Various deformities in apical hair bundles were also detected in P3 neonatal *Daple*^−/−^ mice (Fig. 3A, Supplementary Fig. S2). As for the kinocilia, they were not always in the center of hair bundles (Fig. 3B). Some kinocilia were located at the center of hair bundles, while the bundles were split (Fig. 3B b). To evaluate apical morphological deformities, we classified kinocilia into three groups based on their localization relative to the hair bundle: normal (centered), off-centered, and poorly determined. The term “poorly examined” was used when the position could not be determined as centered or off-centered (Fig. 3B a). The results revealed that > 50% of the kinocilia were localized away from the center of the hair bundles, exhibiting a disrupted correlation between kinocilia and hair bundles (Fig. 3C). In terms of the arrangements of hair bundles, approximately 80% of HCs were abnormal in the basal region of the cochleae, which is the most developmentally mature cochlear region.

### Apical microtubules emanate irregularly from disorderly aggregated structures in murine Daple^−/−^ cochlear hair cells

Prior studies on ependymal cells have demonstrated that Daple functions as a regulator of microtubule polymerization  in mouse ventricles^7^. In cochlear HCs, apical microtubules are also important regulators of the apical morphogenesis of HCs of the OC. In this study, we focused on the apical microtubule networks of HCs of the OC to clarify their roles in apical morphogenesis in *Daple*^−/−^ mice.

**Figure 4:**
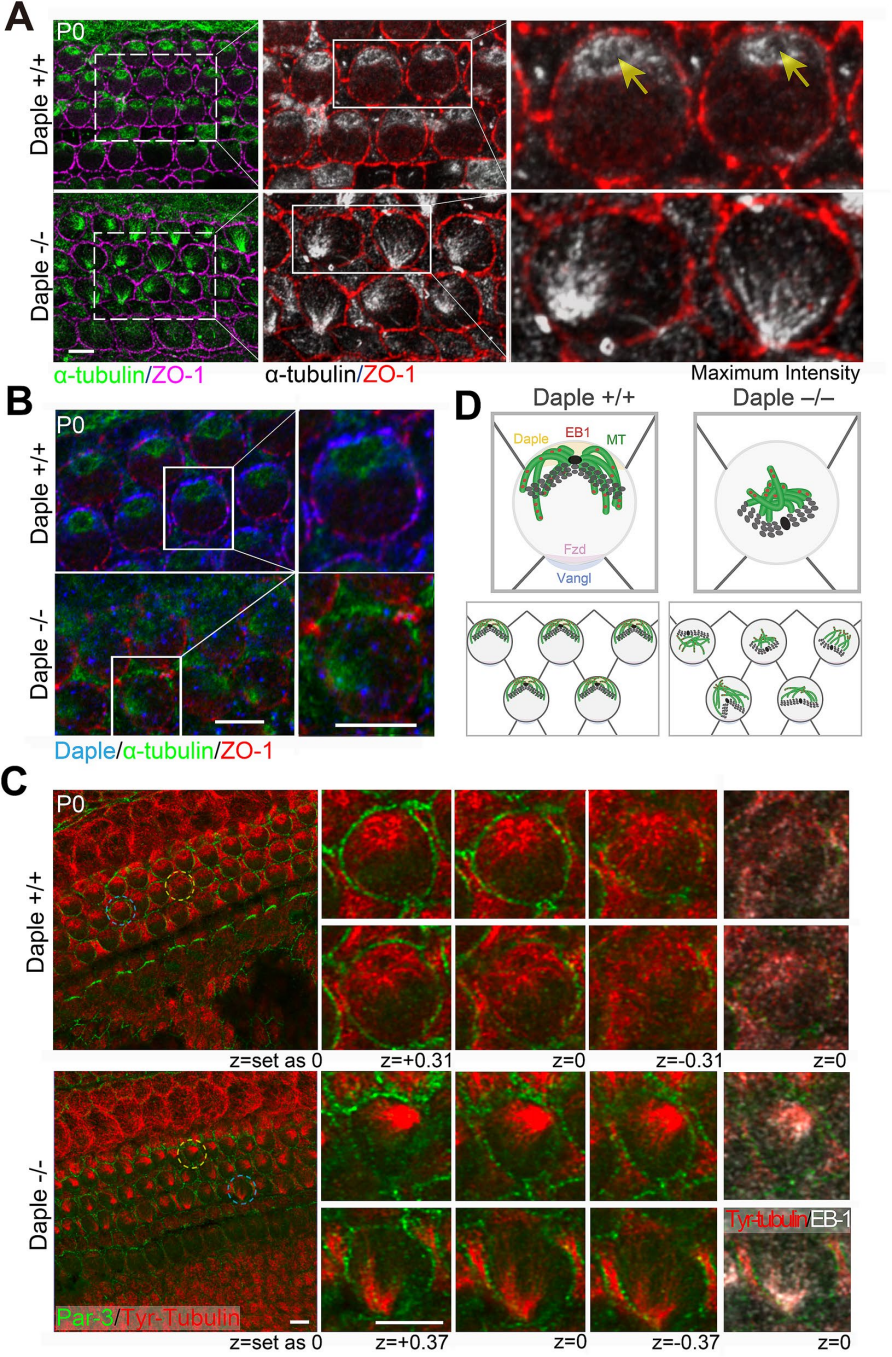
Microtubules and related protein expression in hair cells (HCs) of the organ of Corti (OC) in *Daple*^−/−^ mice. (**A**) Microtubules were spread from the pericentriolar area (arrows) to outer hair cell (OHC) cortexes, mainly in lateral areas in *Daple*^+/+^ P0 mice. Microtubules were disorganized or aggregated around pericentriolar areas in *Daple*^−/−^ P0 mice. (**B**) Daple and microtubule distribution in *Daple*^+/+^ P0 mice. Daple surrounded the microtubules in the centrosomes of the HCs of *Daple*^+/+^ mice. Some non-specific staining was observed, but ring-like staining was not detected in *Daple*^−/−^ HCs. (**C**) We compared a z-series of images against microtubules/EB-1. EB-1 was concentrated in microtubule aggregations in many *Daple*^−/−^HCs. (**D**) Schematic of apical microtubule networks in HCs of Daple WT mice compared to those of Daple KO mice. Scale bars: A, B, C, 5 μm.

In P0 *Daple*^+/+^ HCs, apical microtubules were laterally localized, consistent with the lateral positioning of the kinocilium along the mediolateral axis in HCs. The microtubules spread from the pericentriolar region, the base of the kinocilium, toward the cell cortex, to attach to the lateral side of the plasma membrane (Fig. 4A; *Daple*^+/+^). In contrast, the opposing ends of microtubules formed ring-like structures around the centrosome. In *Daple*^−/−^ HCs, densely aggregated microtubule structures were observed at random positions within the cells, and many microtubules were not attached to the lateral membrane (Fig. 4A). As shown in Fig. 4B in *Daple*^+/+^ HCs, microtubule rings were surrounded by a ring of Daple. The role of Daple in the accurate organization of the microtubule network in the apical region is evident from Fig. 4B. The presence of Daple was confirmed by staining *Daple*^−/−^ and *Daple*^+/+^ HCs of the OC with the same antibody.

**Figure 5:**
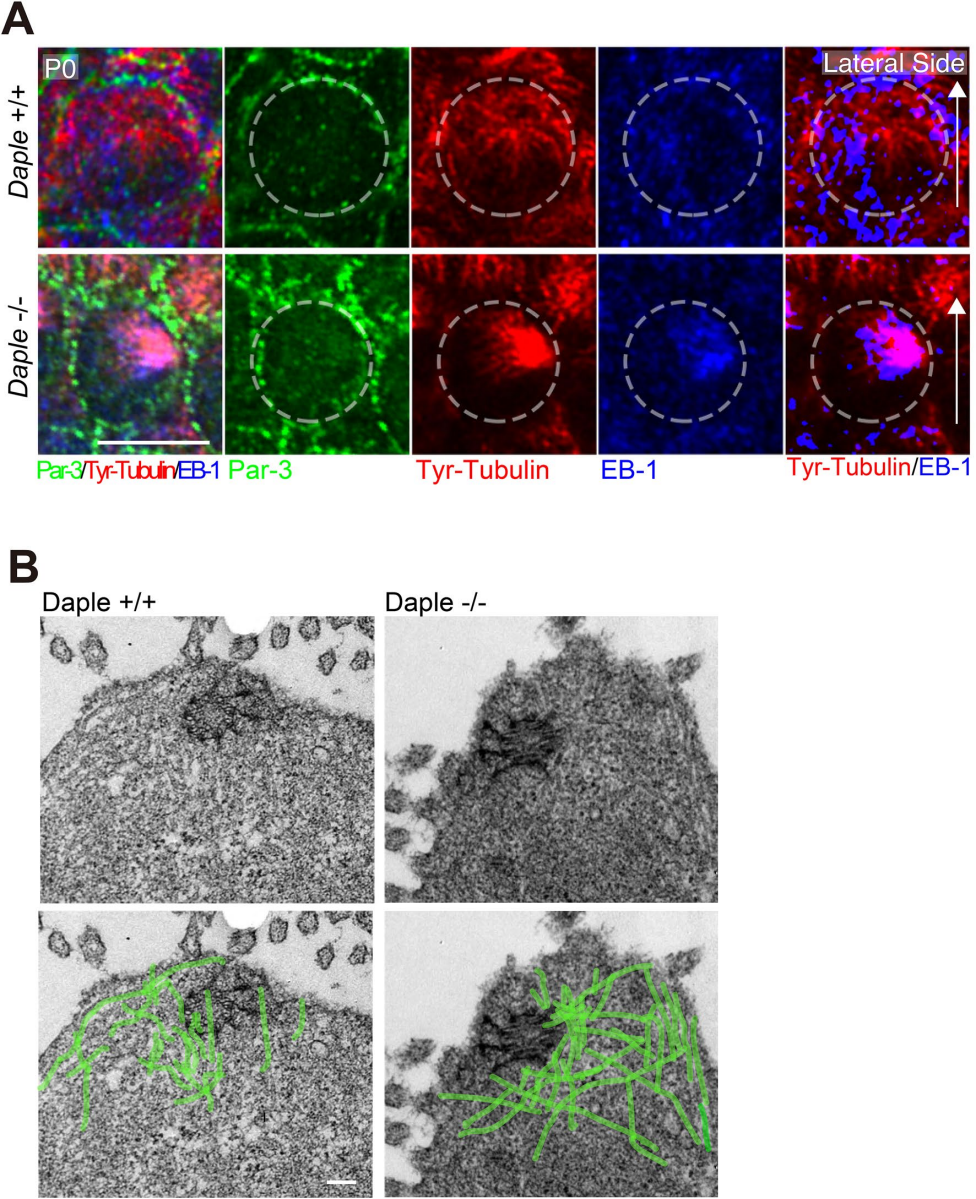
EB-1/Tyr-tubulin staining and transmission electron microscopy (TEM) images. (**A**) These images were generated from Fig. 4C to show the clear distribution difference of microtubules and EB-1 between *Daple*^+*/*+^ and *Daple*^−/−^ mice. The dotted line circle shows the cell border. The rightmost figures, where the EB-1 distribution was binarized using Photoshop software and merged with the staining of Tyr-Tubulin, showing characteristics distribution of Tyr-Tubulin and EB-1. The distribution of EB-1, a microtubule plus-end binding protein, was observed near the lateral membrane of hair cells (HCs) in *Daple*^+/+^ mice. However, EB-1 in *Daple*^−/−^ HCs was more densely aggregated in the cytoplasm. Scale bar: 5 μm. (**B**) The microtubular network extending from the basal body was clearly observed in murine *Daple*^+/+^ HCs. However, the microtubules exhibited a denser distribution around basal bodies in *Daple*^−/−^ mice. Microtubules were identified by their properties, for example, around 25 nm diameter, tube-like high density structure^23^. Scale bar: 1 μm.

To examine the 3D structure of the apical microtubule network in detail, we compared the z-series of images for microtubules/EB-1 (Fig. 4C). In *Daple*^+/+^ mice, the microtubule network, the center of which has a ring-like region, radially and dominantly emanated to the lateral side, whereas smaller amounts of microtubules were diffusely directed to the medial side. The microtubules diffused like an umbrella in *Daple*^+/+^ HC. Upon comparing the distribution of EB-1, a microtubule plus-ended binding protein, to that of microtubule, EB-1 was found to be diffusely distributed through the cytoplasm relatively merged with the distribution of microtubule. In contrast, in murine *Daple*^−/−^ HCs, microtubule were disorderly aggregated, without ring-like regions in the centers of aggregation, and a smaller amount of the microtubule network, diffused within the cytoplasm, was observed compared to that in *Daple*^+/+^ HCs. When z-sliced images were observed, microtubule aggregates were clearly present at very high densities in *Daple*^−/−^ mice. The results are illustrated in Fig. 5A and suggest that Daple plays a role in forming correct microtubule networks in mouse HCs.

### Disordered microtubules were observed to run through the HC apical planes in transmission electron microscopy (TEM) images of Daple^−/−^ mice

To obtain a clearer distribution of the microtubule network, we next performed thin-section TEM (Fig. 5B). In the thin-section TEM images of *Daple*^+/+^ mice, microtubules surrounding the centrosome were clearly observed, but those around the centrosome seemed relatively sparse, suggesting that this area may be the ring-like region observed in immunofluorescence images. Microtubules were found to emanate from the pericentriolar region and attached to the lateral cell membrane in HCs. A smaller number of microtubules seemed to run in the medial direction. In contrast, in *Daple*^−/−^ mice, as evident from the immunofluorescence images (Fig. 4), a higher density of microtubules compared with that in cochleae of *Daple*^+/+^ mice was present around the centrosomes without ring-like structures, forming a sparse region. Furthermore, some microtubules were unnaturally elongated in the cytoplasm. These results showed that the deficiency in Daple induced disturbed microtubule arrays in mice.

### Cochlear hair bundle abnormalities were induced by nocodazole, a microtubule polymerization inhibitor

**Figure 6:**
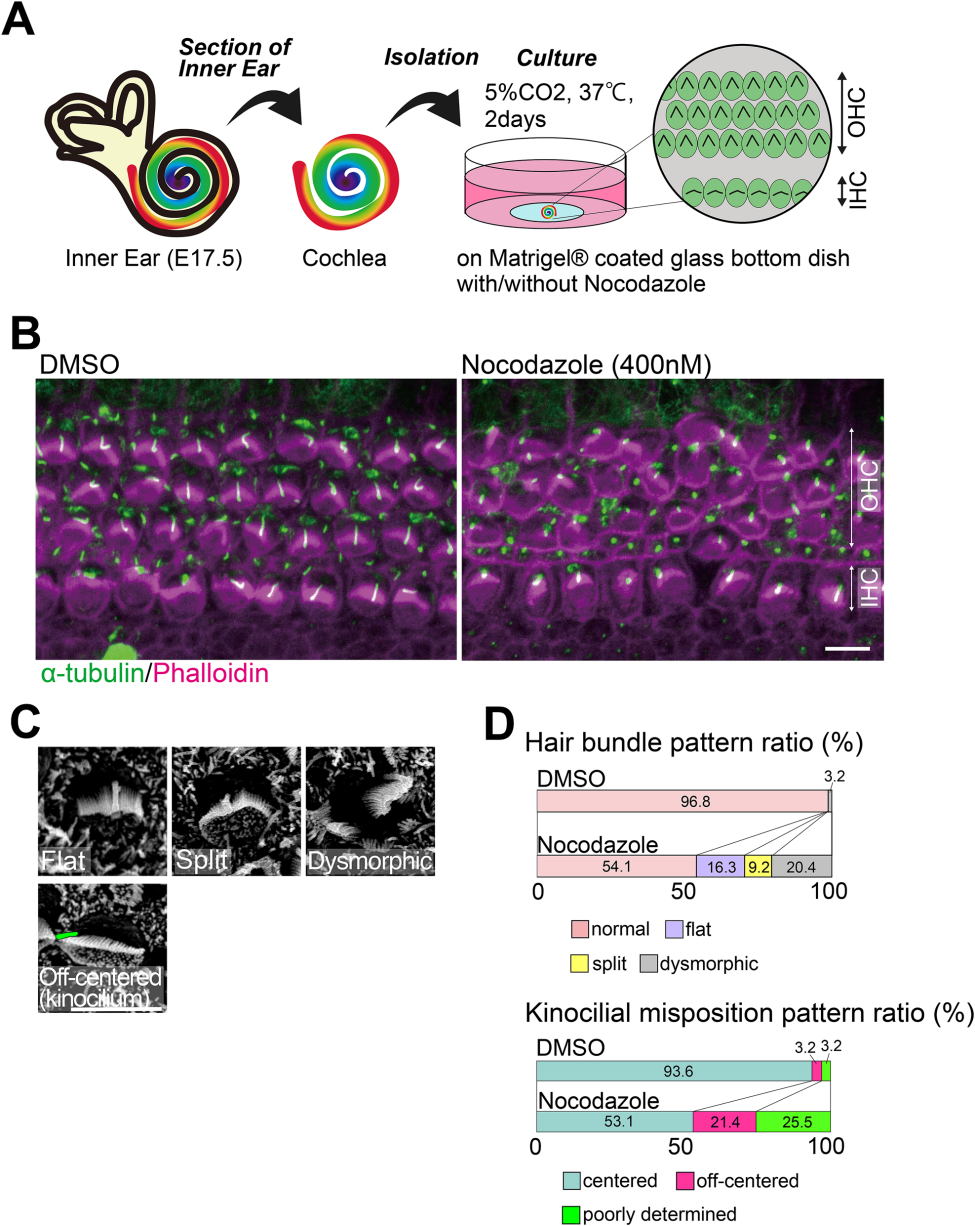
Cochlear organ culture with nocodazole treatment. (**A**) We performed cochlear organ culture starting at embryonic day (E)17.5. (**B**) After treatment with 0.05% DMSO or 400 nM nocodazole for 2 days, we performed immunostaining analyses against α-tubulin (green) and phalloidin (magenta). Many stereocilia bundles exhibited dysmorphic patterns. (**C**) In scanning electron microscopy (SEM) images, some stereocilia exhibited mis-shaped and off-centered patterns after nocodazole treatment. (**D**) To understand differences between the two groups (cochlear organ culture started at E17.5, DMSO 80 cells, nocodazole 98 cells), we counted and summarized them in (**D**).

The abovementioned results suggest that disordered microtubules contribute to the deformity in hair bundles in *Daple*^−/−^ mice. To prove the validity of this hypothesis, we performed nocodazole treatment-based experiments on organ cultures of OC cells (Fig. 6A). The base region of the cochlea from embryonic day (E)17.5 mice was dissected, and the epithelial layer with three arrays of OHCs and one array of inner hair cells (IHC) were mechanically isolated using a stereo microscope for subsequent organ culture. DMSO-treated samples were prepared for use as a control (Fig. 6B; DMSO). Without nocodazole, almost all HCs developed normally. Upon exposure to nocodazole (400 nM) for 2 days at 37 °C in a 5% CO_2_ incubator, stereocilia developed mis-shaped hair bundles, with various kinds of changes, including the presence of flat, dysmorphic, or off-centered kinocilia in HCs, similar to those in *Daple*^−/−^ mice (Fig. 6B,C). Approximately half of the cochlear culture HCs treated with 400 nM nocodazole did not develop correct hair bundles and, instead, had dysmorphic bundle patterns (Fig. 6D). No changes in the expression of PCP core protein were observed under these conditions, suggesting that these results were not related to tissue PCP but to cellular signals (Supplementary Fig. S3).

## Discussion

Here, first, hearing defects across a broad range of frequencies, especially at frequencies > 24 kHz, were found in 8–12-week-old adult *Daple*^−/−^ mice. As sounds with a frequency > 24 kHz can cause approximately 30% of the basal region of the membrane to vibrate^10^, this result was consistent with the observation that more severe defects in hair bundle arrangement were present in more basal areas of the OC, as detected by SEM imaging. By comparing adult and neonatal mice (approximately P3), we also found that defects did not clearly progress in the apical structure of hair cells after the neonatal stage. This suggested that Daple is required for the acquisition of hearing ability in adult stages.

There was a recent report concerning the special role of Gαi_3_ activation through the GBA domain^11^, and mislocalization of apical Gαi_3_ in HCs in Daple-deficient mice^8^. Gαi_3_ mutant mice exhibited more severe mis-shaped hair bundle arrangements in the basal area of the cochlea duct^12^. This led us to speculate Daple deficiency and Gαi_3_ function_,_ may be correlated, specifically in the basal region of the OC.

In ventricular ependymal cells, Daple is reported to function in microtubule dynamics^7^. In previous reports, microtubules were suggested to be important for the apical arrangement of HCs^9^. The conditional knockout of several microtubule-related proteins, such as Lis1, a dynein activating microtubule-binding protein, disturbs the organization of microtubules by impairing developmental stage-specific connections between the microtubules and plasma membranes through the LGN (Gpsm2)–Gαi–dynein complex^9^. This might lead to the formation of an apical microtubule-rich bare zone and the stabilization of Gαi3–Daple–Dvl complexes on the lateral side of the plasma membrane in HCs. Our results regarding the dysregulation of EB1 and focal localization in *Daple*^−/−^ mice also support this notion. This also suggests a close relationship between microtubules and apical structures, including stereocilia/kinocilia morphogenesis. On the other hand, Dvl deficiency induces various degrees of hair bundle malformations because of defects in the combination of Dvl 1–3, as reported previously^13,14^. Disturbance in Dvl subtype-specific interactions with Daple may have induced severe changes in the basal area of *Daple*^−/−^ cochlea.

Several reports have demonstrated that other PCP pathways and actin cytoskeletal dysfunction are involved in various forms of cochlear malformation that are different from those seen in Daple-deficient mice. PCP proteins, such as Vangl, have a normal V-shape, but disorientated, hair bundle arrangements that are different from those in *Daple*^−/−^ mice^15^. As for actin, it would be informative to investigate Rho-family protein-deficient mice, because of the upstream regulatory role of Rho-family proteins in the organization of actin filaments. Rac1-deficient mice have defects in the arrangement of hair bundles similar to those in Daple-deficient mice, but these are different from those in *Daple*^−/−^ mice in that the cochlear duct is significantly shortened and the fragmentation of hair bundles is severely progressive around birth^16^. Moreover, the cochlear abnormalities observed in *Daple*^−/−^ mice were different from those in mice deficient in actomyosin-related proteins, such as myosin2^17^, RhoA^18^, and Cdc42^19,20^.

In conclusion, we validated the hypothesis that Daple regulates the organization of microtubules in HCs of the OC, as well as in ependymal cells. E17.5 mouse cochlear organ culture revealed that the defects in the arrangements of stereocilia bundles after treatment with nocodazole were similar to those in Daple-deficient mice. This is the first study regarding cochlear HC differentiation employing microtubule polymerization inhibitors, except for the examination of apical surface rigidity^21^. Further studies examining cochlear cytoskeletal maturation processes in shorter intervals around birth may reveal the sequence of underlying molecular mechanisms and their related signals.

## Methods

All methods were conducted in accordance with ARRIVE guidelines.

### Ethics statement

Animal experiments were performed in accordance with protocols approved by the animal studies committee of Osaka University, School of Medicine and Frontier Biosciences. Recombinant DNA experiments were carried out in accordance with the protocols approved by Osaka University.

### Generation of Daple-deficient mice

We used a targeted ESC clone (DEPD00564-1-G07) from the trans-NIH KOMP Repository (University of California, Davis) to generate Daple mutant mice as previously reported^7^. Animal care and use was in accordance with the Guidelines for Proper Conduct of Animal Experiments in Osaka University and was approved by the Animal Care and Use Committee at Osaka University.

### Auditory brainstem response (ABR) test

The details of the ABR test and the method have been reported previously^22^. We injected ketamine (100 mg/kg) and xylazine (10 mg/kg) into the peritoneal cavity of mice and put mice into a sound isolation chamber. Subcutaneous needle electrodes were inserted in the pinna and vertex, with a ground electrode near the tail. Responses to tone pip stimuli were recorded at 4, 8, 12, 24, and 32 kHz, intensities ranging from 0 to 100 dB-SPL instep of 5 dB, in 8–10-week-old mice using a Power Lab 2/25 (AD Instruments, Australia) and a TDT Auditory Workstation (Tucker-Davis Technologies, Alachua, Florida, USA). The duration of tone bursts was 1 ms. We amplified and averaged 500 responses. All ABRs were measured without knowing the profiles or genotypes of the mice.

### Assessment of Daple gene expression by X-gal staining

Inner ears obtained from Daple + /- and Daple + / + adult mice were fixed in 4% paraformaldehyde (PFA) in PBS for 15–30 min. Staining was performed for 48 h at 37 °C in 2 mg/mL X-gal (Promega)/2 mM MgCl_2_/0.02% NP40/0.01% sodiumdeoxycholate/5 mM K_4_Fe(CN)_6_/5 mM K_3_Fe(CN)_6_ in PBS.

### Immunofluorescence staining

Inner ears obtained from Daple + / + or Daple-/-mice were dissected from adult mice or pups and fixed in 4% PFA in PBS at 4 °C overnight, or in absolute methanol at − 20 °C for 10–20 min, or in 10% TCA on ice for 1 h. After fixation in 4% PFA or absolute methanol, adult inner ears were decalcified in EDTA. After fixation of the inner ears by PFA, they were permeabilized with 0.15% Triton X-100 in PBS at room temperature (RT) for 15 min. Whole mount organs were blocked for 1 h with 10% bovine serum albumin in PBS or with the blocking reagent (M.O.M.™; Vector Laboratories, Inc.). They were then incubated with primary antibodies at 4 °C overnight and washed three times with PBS. Staining was performed with Alexa Fluor-conjugated secondary antibodies at RT for 1 h. The following primary antibodies were used: monoclonal anti-mouse α-tubulin antibody (T9026; Sigma-Aldrich) 1:500; anti-rabbit Daple antibody (28,147; IBL) 1:100; anti-rat ZO-1 antibody (PA5-18,646; Thermo fisher) 1:400; anti-rat tyrosinated alpha-tubulin (ab6160; Abcam) 1:500; anti-mouse EB-1 (610,535; BD) 1:500; anti-goat Frizzled 6 (AF1526; R&D Systems) 1:200; and anti-rabbit Par-3 (07–330; Sigma-Aldrich) 1:500. The following secondary antibodies were used: Alexa Fluor 488-conjugated donkey anti-mouse IgG (Jackson Immuno Research) 1:500; Cy3-conjugated donkey anti-rat IgG, Alexa Fluor 647-conjugated donkey anti-rabbit IgG; Alexa Fluor 647-conjugated donkey anti goat IgG; and rhodamine phalloidin (Cytoskeleton, Inc.) 1:500. Images were collected using a Zeiss LSM 710 or LSM 880 confocal microscope, and the obtained images were analyzed with the Zen Software.

### Scanning electron microscopy (SEM)

Inner ears obtained from Daple + / + or Daple-/- mice were fixed with 2% PFA and 2.5% glutaraldehyde in 0.1 M HEPES (pH 7.4) for 1 h at RT. They were then washed with 0.1 M HEPES and fixed in 1% OsO4 for 1 h on ice, incubated in 1% tannic acid overnight, and fixed in 1% OsO4 for 1 h on ice again. The organ of Corti was micro-dissected, dehydrated, dried at the critical point, sputter-coated, and observed using SEM (S-4800 microscope; Hitachi).

### Transmission electron microscopy (TEM)

Inner ears obtained from Daple + / + or Daple-/- mice were fixed with 2% PFA and 2.5% glutaraldehyde and treated with 2% tannic acid in 0.1 M HEPES (pH 7.4) for 1 h at RT. They were then washed with 0.1 M HEPES and fixed in 1% OsO4 for 2 h on ice. The organ of Corti was micro-dissected, dehydrated, embedded, sectioned, and observed using TEM (JEM-1400Plus; JEOL). Microtubules were identified by their properties, for example, around 25 nm diameter, tube-like high density structure^23^.

### Culture of embryonic mouse cochlea and drug treatment

Cochlear organ culture was started from E17.5 mice. Briefly, cochleae were dissected in Leibobitz L-15 medium (Thermo Fisher Scientific) and established on coverslips coated with Matrigel matrix (Corning). Explants were then maintained for 1 h in vitro in DMEM/F-12 (Invitrogen) supplemented with FBS and ampicillin (Nacalai Tesque). Next, the medium was replaced with that containing DMSO only (control) or nocodazole (Sigma; 400 and 800 nM). After 2 days of culture in vitro, the explants were fixed with 4% PFA or methanol for immunostaining, or with 2% PFA plus 2.5% glutaraldehyde in 0.1 M HEPES (pH 7.4) for SEM.

### Statistical analysis

The statistical data in Fig. 1A are expressed as mean ± SEM. Comparisons were performed using Mann–Whitney’s test, and differences with *p* < 0.05 were considered statistically significant.

Figures and images were prepared using Zen 3.0 SR FP1 (black) (https://www.zeiss.co.jp/microscopy/downloads.html; Carl Zeiss Microsystems) and Adobe Photoshop 2020 and Illustrator 2020 (https://www.adobe.com/jp/products/photoshop.html, https://www.adobe.com/jp/products/illustrator.html; Adobe Systems Inc.).

## Supplementary Information

Supplementary Information.
