# Supplementary material for: Daple deficiency causes hearing loss in adult mice by inducing defects in cochlear stereocilia and apical microtubules
Source: Sci Rep. 2021 Oct 12;11:20224. doi: 10.1038/s41598-021-96232-8 (PMC8511111; doi:10.1038/s41598-021-96232-8)
Supplement: Supplementary file 1 — Supplementary Information. [file 41598_2021_96232_MOESM1_ESM.pdf]

Supplementary Information

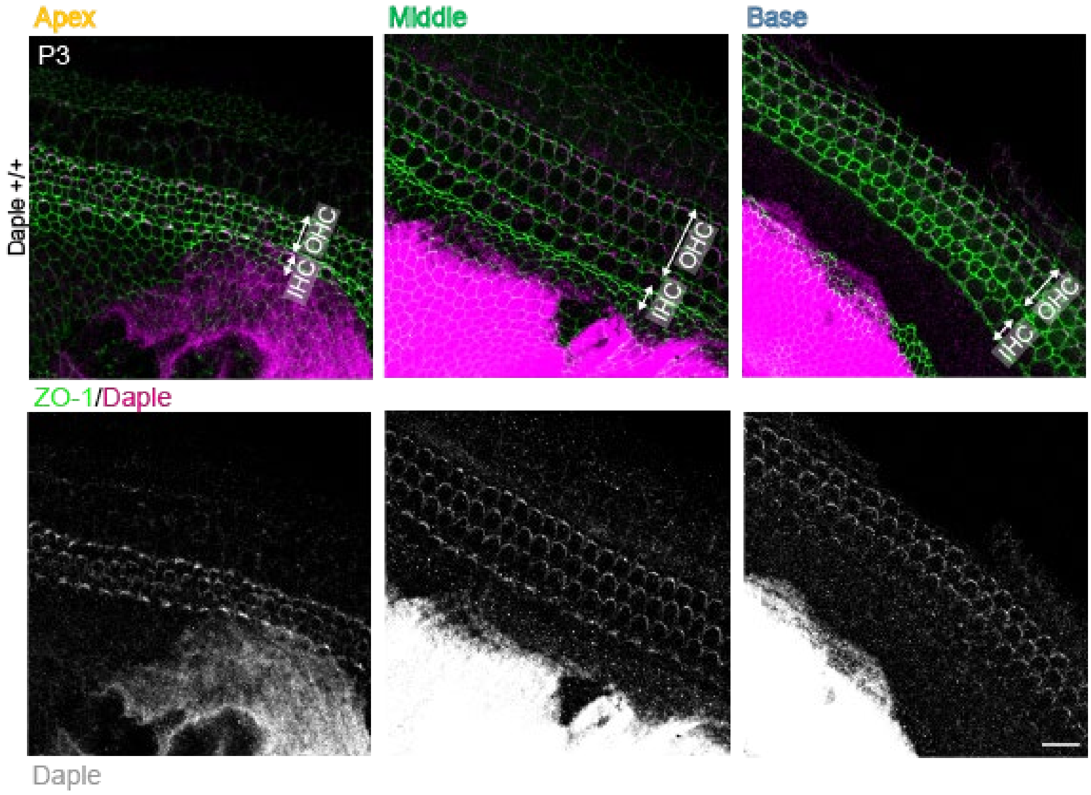

Fig. S1 Localization of Daple in cochlea epithelial cells in P3 mice.

Daple was localized in the apex, middle, and basal areas of the organ of Corti (OC). As shown in the images, Daple was expressed on the lateral side of hair cells. Scale bar: 10 µm.

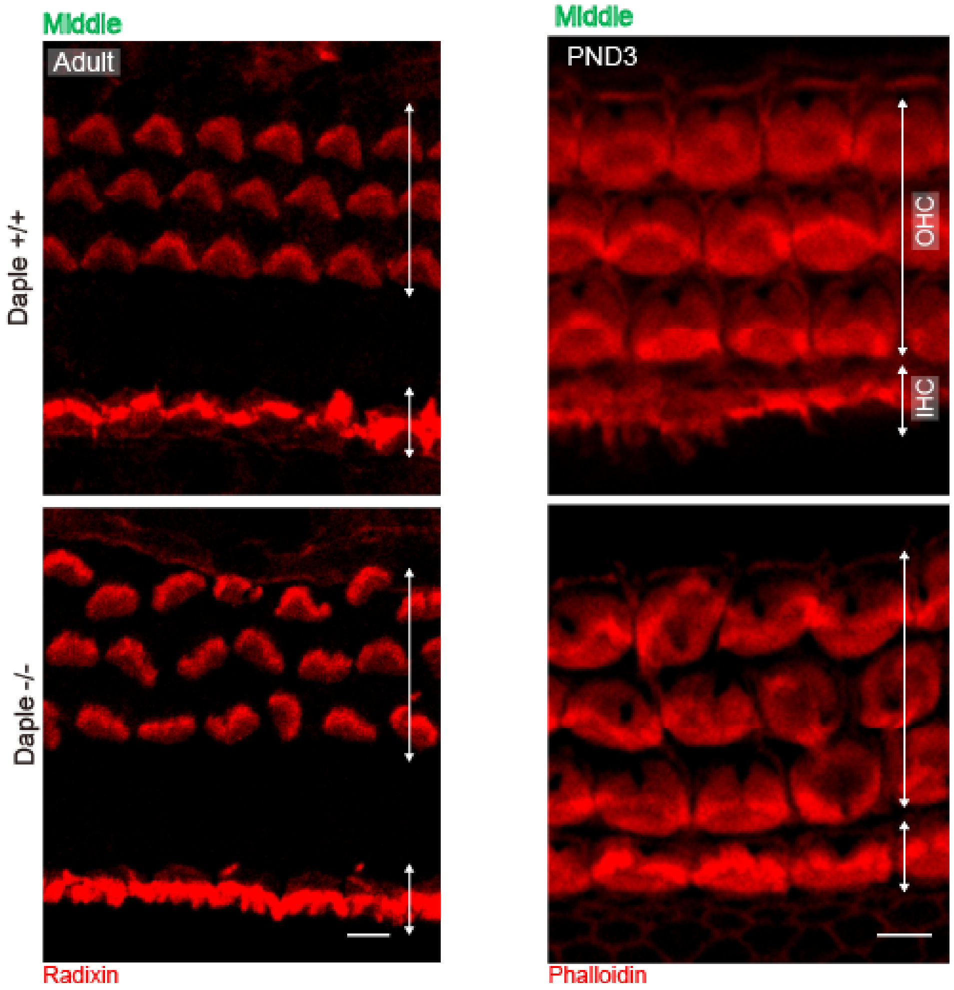

Fig. S2 Distribution and expression of actin and actin related protein.

Morphological changes in hair bundles in *Daple*$^{-/-}$ hair cells (HCs) compared to those in *Daple*$^{+/+}$ HCs visualized by staining of radixin at adult and actin at P3 stages. However, the expression levels of actin and actin-related proteins were unchanged in the adult and P3 cochlea. Scale bars: 5 μm.

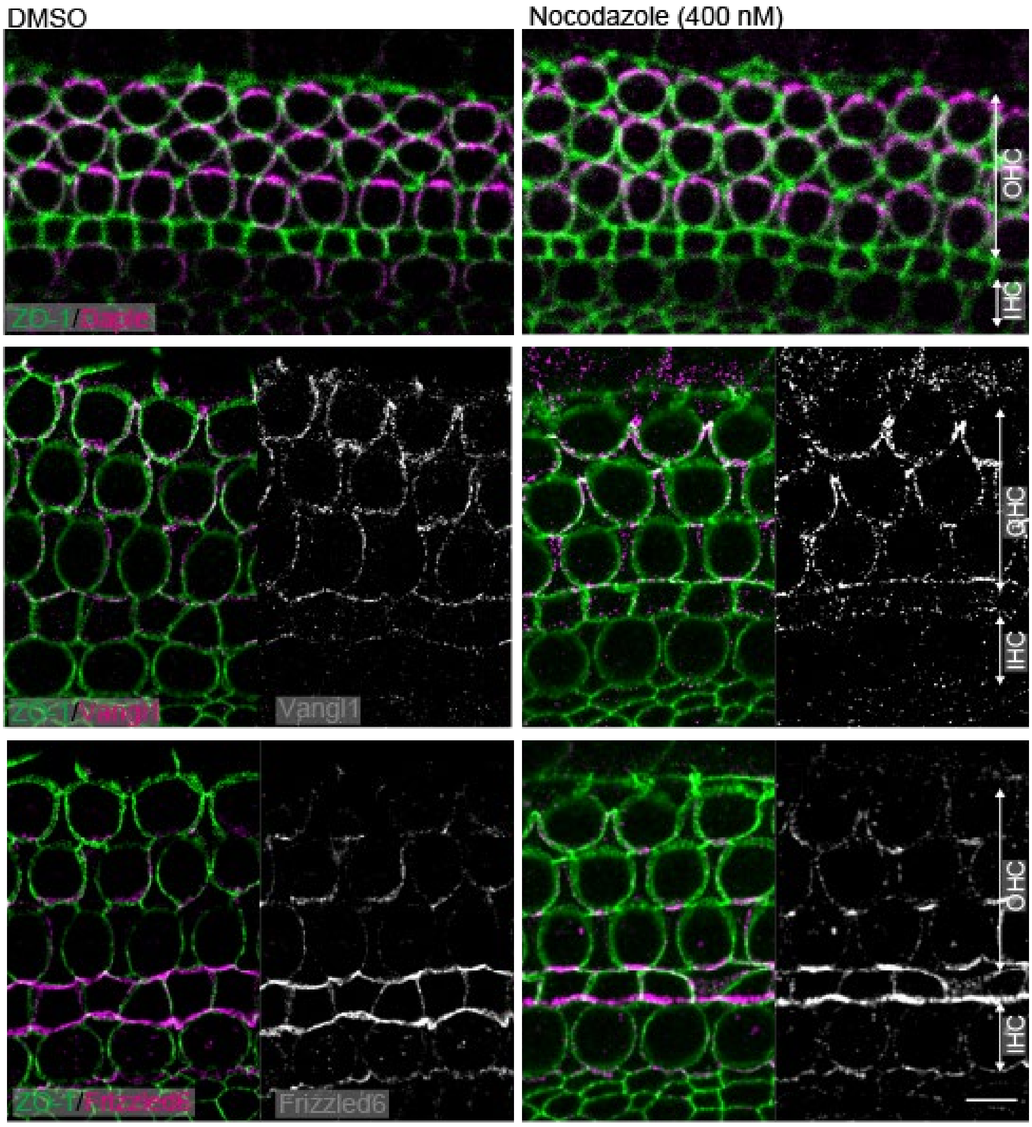

Fig.S3 Distribution and expression of core PCP proteins.

The distribution and expression of Vangl1 and Frizzled6, core PCP proteins after nocodazole treatment were similar to those in non-treated cells. Scale bar: 5 μm.
